# Improving the Accuracy of the Fast Inverse Square Root by Modifying Newton–Raphson Corrections

**DOI:** 10.3390/e23010086

**Published:** 2021-01-09

**Authors:** Cezary J. Walczyk, Leonid V. Moroz, Jan L. Cieśliński

**Affiliations:** 1 Wydział Fizyki, Uniwersytet w Białymstoku, ul. Ciołkowskiego 1L, 15-245 Białystok, Poland; c.walczyk@uwb.edu.pl; 2 Department of Security Information and Technology, Lviv Polytechnic National University, st. Kn. Romana 1/3, 79000 Lviv, Ukraine; moroz_lv@polynet.lviv.ua

**Keywords:** approximation of functions, floating-point arithmetic, Newton–Raphson method, inverse square root, magic constant

## Abstract

Direct computation of functions using low-complexity algorithms can be applied both for hardware constraints and in systems where storage capacity is a challenge for processing a large volume of data. We present improved algorithms for fast calculation of the inverse square root function for single-precision and double-precision floating-point numbers. Higher precision is also discussed. Our approach consists in minimizing maximal errors by finding optimal magic constants and modifying the Newton–Raphson coefficients. The obtained algorithms are much more accurate than the original fast inverse square root algorithm and have similar very low computational costs.

## 1. Introduction

Efficient performance of algebraic operations in the framework of floating-point arithmetic is a subject of considerable importance [1,2,3,4,5,6]. Approximations of elementary functions are crucial in scientific computing, computer graphics, signal processing, and other fields of engineering and science [7,8,9,10]. Our aim is to compute elementary functions at a very low computational cost without using memory resources. Direct evaluation of functions could be of interest in any systems where storage capabilities challenge the processing of a large volume of data. This problem is crucial, for instance, in high-energy physics experiments [11,12,13].

In this paper, we consider approximation and fast computation of the inverse square root function, which has numerous applications (see [8,10,14,15,16,17]), especially in 3D computer graphics, where it is needed for normalization of vectors [4,18,19]. The proposed algorithms are aimed primarily at floating-point platforms with limited hardware resources, such as microcontrollers, some field-programmable gate arrays (FPGAs), and graphics processing units (GPUs) that cannot use fast look-up table (LUT)-based hardware instructions, such as SSE (i.e., Streaming SIMD (single instruction, multiple data) Extensions) or Advanced Vector Extensions (AVX). We mean here devices and chips containing floating-point multipliers, adders–subtractors, and fused-multiply adders. Therefore, our algorithms can easily be implemented on such a platform. We also offer them as an alternative to library functions that provide full precision, but are very time consuming. This was the motivation for considering the cases of higher precision in Section 3.2. By selecting the precision and number of iterations, the desired accuracy can be obtained. We propose the use of our codes as direct insertions into more general algorithms without referring to the corresponding library of mathematical functions. In the double-precision mode, most modern processors do not have SSE instructions like rsqrt (such instructions appeared only in AVX-512, which is supported only by the latest processor models). In such cases, one can use our algorithms (with the appropriate number of iterations) as a fast alternative to the library function 1/sqrt(x).

In most cases, the initial seed needed to start the approximation is taken from a memory-consuming look-up table (LUT), although the so-called “bipartite table methods” (actually used on many current processors) make it possible to considerably lower the table sizes [20,21]. The “fast inverse square root” code works in a different way. It produces the initial seed in a cheap and effective way using the so-called magic constant [4,19,22,23,24,25]. We point out that this algorithm is still useful in numerous software applications and hardware implementations (see, e.g., [17,26,27,28,29,30]). Recently, we presented a new approach to the fast inverse square root code *InvSqrt*, presenting a rigorous derivation of the well-known code [31]. Then, this approach was used to construct a more accurate modification (called *InvSqrt1*) of the fast inverse square root (see [32]). It will be developed and generalized in the next sections, where we will show how to increase the accuracy of the *InvSqrt* code without losing its advantages, including the low computational cost. We will construct and test two new algorithms, *InvSqrt2* and *InvSqrt3*.

The main idea of the algorithm *InvSqrt* consists in interpreting bits of the input floating-point number as an integer [31]. In this paper, we consider positive floating-point normal numbers

(1) $$x=\left(1+m_{x}\right)2^{e_{x}}\;,\;m_{x}\in \left[0,1\right)\;,\;e_{x}\in Z\;,$$

and, in Section 3.1, we also consider subnormal numbers. We use the standard IEEE-754, where single-precision floating-point numbers are encoded with 32 bits. For positive numbers, the first bit is zero. The next eight bits encode $e_{x}$, and the remaining 23 bits represent the mantissa $m_{x}$. The same 32 bits can be treated as an integer $I_{x}$:

(2) $$I_{x}=N_{m}\left(B+e_{x}+m_{x}\right),$$

where $N_{m}=2^{23}$ and B=127. In this case $B+e_{x}$ is a natural number not exceeding 254. The case of higher precision is analogous (see Section 3.2).

The crucial step of the algorithm *InvSqrt* consists in shifting all bits to the right by one bit and subtracting the result of this operation from a “magic constant” *R* (and the optimum value of *R* has to be guessed or determined). In other words,

(3) $$I_{y_{0}}=R-\left\lfloor I_{x}/2\right\rfloor \;.$$

Originally, *R* was proposed as 0x5F3759DF (see [19,23]). Interpreted in terms of floating-point numbers, $I_{y_{0}}$ approximates the inverse square root function surprisingly well ($y_{0}\approx y=1/\sqrt{x}$). This trick works because (3) is close to dividing the floating-point exponent by −2. The number *R* is needed because the floating-point exponents are biased (see (2)).

The magic constant *R* is usually given as a hexadecimal integer. The same bits encode the floating-point number $R_{f}$ with an exponent $e_{R}$ and mantissa $m_{R}$. According to (1), $R_{f}=\left(1+m_{R}\right)2^{e_{R}}$. In [31], we have shown that if $e_{R}=\frac{1}{2}\left(B-1\right)$ (e.g., $e_{R}=63$ in the 32-bit case), then the function (3) (defined on integers) is equivalent to the following piece-wise linear function (when interpreted in terms of corresponding floating-point numbers):

(4) $${\tilde{y}}_{0}\left(\tilde{x},t\right)=\left\{\begin{array}{cc} \frac{1}{8}\left(6-2\tilde{x}+t\right) & \;\mathrm{for}\;\;\tilde{x}\in \left[1,2\right)\\ \frac{1}{8}\left(4+t-\tilde{x}\right) & \;\mathrm{for}\;\;\tilde{x}\in \left[2,t\right)\\ \frac{1}{16}\left(8+t-\tilde{x}\right) & \;\mathrm{for}\;\;\tilde{x}\in \left[t,4\right) \end{array}\right.$$

where

(5) $$t=2+4m_{R}+2\mu_{\tilde{x}}N_{m}^{-1},$$

$m_{R}$ is the mantissa of *R* (i.e., $m_{R}:=N_{m}^{-1}R-\left\lfloor N_{m}^{-1}R\right\rfloor$), and, finally, $\mu_{\tilde{x}}=0$ or $\mu_{\tilde{x}}=1$ depending on the parity of the last digit of the mantissa of $\tilde{x}$.

The function $\mu_{\tilde{x}}$ is two-valued, so a given parameter *t* may correspond to either two values of *R* or one value of *R* (when the term containing $\mu_{\tilde{x}}$ has no influence on the bits of the mantissa $m_{R}$). The function $y=1/\sqrt{x}$, the function (3), and all Newton–Raphson corrections considered below are invariant under the scaling $\tilde{x}=2^{-2n}x$ and $\tilde{y}=2^{n}y$ for any integer *n*. Therefore, we can confine ourselves to numbers from the interval [1,4). Here and in the following, the tilde always denotes quantities defined on the interval [1,4).

In this paper, we focus on the Newton–Raphson corrections, which form the second part of the *InvSqrt* code. Following and developing ideas presented in our recent papers [31,32], we propose modifications of the Newton–Raphson formulas, which result in algorithms that have the same or similar computational cost as/to *InvSqrt*, but improve the accuracy of the original code, even by several times. The modifications consist in changing both the Newton–Raphson coefficients and the magic constant. Moreover, we extend our approach to subnormal numbers and to higher-precision cases.

## 2. Modified Newton–Raphson Formulas

The standard Newton–Raphson corrections ${\tilde{y}}_{1}$ and ${\tilde{y}}_{2}$ for the zeroth approximation ${\tilde{y}}_{0}$ given by (4) are given by the following formulas:

(6) $$\begin{array}{c} {\tilde{y}}_{1}\left(\tilde{x},t\right)=\frac{3}{2}{\tilde{y}}_{0}\left(\tilde{x},t\right)-\frac{1}{2}\tilde{x}\;{\tilde{y}}_{0}^{3}\left(\tilde{x},t\right)\;,\\ {\tilde{y}}_{2}\left(\tilde{x},t\right)=\frac{3}{2}{\tilde{y}}_{1}\left(\tilde{x},t\right)-\frac{1}{2}\tilde{x}\;{\tilde{y}}_{1}^{3}\left(\tilde{x},t\right)\;, \end{array}$$

(analogous formulas hold for the next corrections as well; see [31]). The relative error functions ${\tilde{\delta }}_{j}\left(\tilde{x},t\right)$ (where j=0,1,2,…) can be expressed as:

(7) $${\tilde{\delta }}_{j}\left(\tilde{x},t\right)=\sqrt{\tilde{x}}\;{\tilde{y}}_{j}\left(\tilde{x},t\right)-1\;.$$

The function ${\tilde{\delta }}_{0}\left(\tilde{x},t\right)$, which is very important for the further analysis, is thoroughly described and discussed in [31]. Using (7), we substitute ${\tilde{y}}_{j}=\left(1+{\tilde{\delta }}_{j}\right)/\sqrt{\tilde{x}}$ (for j=0,1,2,…) into (6), $\tilde{x}$ cancels out, and the formulas (6) assume the following form:

(8) $${\tilde{\delta }}_{j}=-\frac{1}{2}{\tilde{\delta }}_{j-1}^{2}\left(3+{\tilde{\delta }}_{j-1}\right)\;,\;\left(j=1,2,\ldots \right),$$

where ${\tilde{\delta }}_{j}={\tilde{\delta }}_{j}\left(\tilde{x},t\right)$. We immediately see that every correction increases the accuracy, even by several orders of magnitude (due to the factor ${\tilde{\delta }}_{j-1}^{2}$). Thus, a very small number of corrections is sufficient to reach the machine precision (see the end of Section 4).

The above approximations depend on the parameter *t* (which can be expressed by the magic constant *R*, see (5)). The best approximation is obtained for $t=t_{k}$ minimizing $\left|\right|{\tilde{\delta }}_{k}\left(t\right)\left|\right|$, i.e.,

(9) $$\left|\right|{\tilde{\delta }}_{k}\left(t_{k}\right)\left|\right|=\underset{t\in (2,4)}{\inf}\left|\right|{\tilde{\delta }}_{k}\left(t\right)\left|\right|\equiv \underset{t\in (2,4)}{\inf}\left(\underset{\tilde{x}\in \left[1,4\right)}{\sup}\left|{\tilde{\delta }}_{k}\left(\tilde{x},t\right)\right|\right)\;.$$

In this paper we confine ourselves to the case $t=t_{1}$ (i.e., we assume $t_{2}=t_{1}$) because the more general case (where the magic constant is also optimized with respect to the assumed number of iterations) is much more cumbersome, and the related increase in accuracy is negligible. Then, we get

(10) $$t_{1}^{\left(0\right)}\approx 3.7298003,\;R^{\left(0\right)}=0x5F375A86,$$

for details, see [31]. The theoretical relative errors are given by

(11) $$\Delta_{1\max}^{\left(0\right)}\equiv \left|\right|{\tilde{\delta }}_{1}\left(t_{1}\right)|\approx 1.75118\cdot 10^{-3},\;\Delta_{2\max}^{\left(0\right)}\equiv \left|\right|{\tilde{\delta }}_{2}\left(t_{1}\right)|\approx 4.60\cdot 10^{-6}.$$

The superscript (0) indicates values corresponding to the algorithm *InvSqrt* (other superscripts will denote modifications of this algorithm).

The idea of increasing the accuracy by a modification of the Newton–Raphson formulas is motivated by the fact that ${\tilde{\delta }}_{k}\left(\tilde{x},t\right)⩽0$ for any $\tilde{x}$ (see [31,32]). Therefore, we can try to shift the graph of ${\tilde{\delta }}_{1}$ upwards (making it more symmetric with respect to the horizontal axis). Then, the errors of the first correction are expected to decrease twice and the errors of the second correction are expected to decrease by about eight times (for more details, see [32]). Indeed, according to (8), reducing the first correction by a factor of 2 will reduce the second correction by a factor of 4. The second correction is also non-positive, so we may shift the graph of ${\tilde{\delta }}_{2}$, once more improving the accuracy by the factor of 2. This procedure can be formalized by postulating the following modification of the Newton–Raphson formulas (6):

(12) $$\begin{array}{c} {\tilde{y}}_{1}=\frac{1}{2}{\tilde{y}}_{0}\left(3-{\tilde{y}}_{0}^{2}\;\tilde{x}\right)+\frac{1}{2}d_{1}\left(a_{1}{\tilde{y}}_{0}+b_{1}{\tilde{y}}_{1}\right),\\ {\tilde{y}}_{2}=\frac{1}{2}{\tilde{y}}_{1}\left(3-{\tilde{y}}_{1}^{2}\;\tilde{x}\right)+\frac{1}{2}d_{2}\left(a_{2}{\tilde{y}}_{1}+b_{2}{\tilde{y}}_{2}\right), \end{array}$$

where $a_{k}+b_{k}=1$ for k=1,2. Thus, we have four independent parameters ($d_{1}$, $d_{2}$, $a_{1}$, and $a_{2}$) to be determined. In other words,

(13) $$\begin{array}{c} {\tilde{y}}_{1}=c_{11}\;{\tilde{y}}_{0}-c_{21}\;\tilde{x}\;{\tilde{y}}_{0}^{3},\\ {\tilde{y}}_{2}=c_{12}\;{\tilde{y}}_{1}-c_{22}\;\tilde{x}\;{\tilde{y}}_{1}^{3}, \end{array}$$

where four coefficients $c_{jk}$ can be expressed by the four coefficients $a_{k}$ and $d_{k}$:

(14) $$c_{1k}=\frac{3+a_{k}d_{k}}{2-\left(1-a_{k}\right)d_{k}}\;,\;c_{2k}=\frac{1}{2-\left(1-a_{k}\right)d_{k}}\;\left(k=1,2\right)\;.$$

We point out that the Newton–Raphson corrections and any of their modifications of the form (13) are obviously invariant with respect to the scaling mentioned at the end of Section 1. Therefore, we can continue to confine our analysis to the interval [1,4).

Below, we present three different algorithms (*InvSqrt1*, *InvSqrt2*, *InvSqrt3*) constructed along the above principles (the last two of them are first introduced in this paper). They will be denoted by superscripts in parentheses, e.g., ${\tilde{y}}_{k}^{\left(N\right)}$ means the *k*th modified Newton–Raphson correction to the algorithm *InvSqrt N*. We always assume that the zeroth approximation is given by (4), i.e.,

(15) $${\tilde{y}}_{0}^{\left(N\right)}={\tilde{y}}_{0}\;\left(N=1,2,3\right)\;,$$

and relative error functions, $\Delta_{j}^{\left(N\right)}$, are expressed as

(16) $$\Delta_{j}^{\left(N\right)}\left(\tilde{x},t\right)=\sqrt{\tilde{x}}\;{\tilde{y}}_{j}^{\left(N\right)}\left(\tilde{x},t\right)-1\;.$$

We point out that the coefficients of our algorithms are obtained without taking rounding errors into account. This issue will be shortly discussed at the end of Section 4.

### 2.1. Algorithm InvSqrt1

Assuming $a_{1}=a_{2}=0$ and $b_{1}=b_{2}=1$, we transform (12) into

(17) $$\begin{array}{c} {\tilde{y}}_{1}^{\left(1\right)}=\frac{1}{2}{\tilde{y}}_{0}^{\left(1\right)}\left(3-{\left({\tilde{y}}_{0}^{\left(1\right)}\right)}^{2}\;\tilde{x}\right)+\frac{1}{2}d_{1}{\tilde{y}}_{1}^{\left(1\right)},\\ {\tilde{y}}_{2}^{\left(1\right)}=\frac{1}{2}{\tilde{y}}_{1}^{\left(1\right)}\left(3-{\left({\tilde{y}}_{1}^{\left(1\right)}\right)}^{2}\;\tilde{x}\right)+\frac{1}{2}d_{2}{\tilde{y}}_{2}^{\left(1\right)}. \end{array}$$

Therefore, ${\tilde{y}}_{1}^{\left(1\right)}$ and ${\tilde{y}}_{2}^{\left(1\right)}$ depend on $\tilde{x}$, *t*, $d_{1}$, and $d_{2}$. Parameters $t=t_{1}^{\left(1\right)}$ and $d_{1}=d_{1}^{\left(1\right)}$ are determined by minimization of $\left|\right|\Delta_{1}^{\left(1\right)}\left(\tilde{x},t\right)\left|\right|$. Then, the parameter $d_{2}=d_{2}^{\left(1\right)}$ is determined by minimization of $\left|\right|\Delta_{2}^{\left(1\right)}\left(\tilde{x},t_{1}^{\left(1\right)}\right)\left|\right|$ (for details, see [32]). As a result, we get:

(18) $$d_{1}^{\left(1\right)}\approx 1.75118\cdot 10^{-3}\;,\;d_{2}^{\left(1\right)}\approx 1.15234\cdot 10^{-6}\;,$$

and $t_{1}^{\left(1\right)}=t_{1}^{\left(0\right)}$ (see (10)). Therefore, $R^{\left(1\right)}=R^{\left(0\right)}$, i.e., *InvSqrt1* has the same magic constant as *InvSqrt*. The theoretical relative errors are given by

(19) $$\begin{array}{c} \Delta_{1\max}^{\left(1\right)}\equiv \left|\right|\Delta_{1}^{\left(1\right)}\left(\tilde{x},t_{1}^{\left(1\right)}\right)\left|\right|\approx 0.87636\cdot 10^{-3},\\ \Delta_{2\max}^{\left(1\right)}\equiv \left|\right|\Delta_{2}^{\left(1\right)}\left(\tilde{x},t_{1}^{\left(1\right)}\right)\left|\right|\approx 5.76\cdot 10^{-7}. \end{array}$$

The algorithm (17) can be written in the form (13), where:

(20) $$c_{1k}^{\left(1\right)}=\frac{3}{2-d_{k}^{\left(1\right)}}\;,\;c_{2k}^{\left(1\right)}=\frac{1}{2-d_{k}^{\left(1\right)}}\;\left(k=1,2\right)\;.$$

Taking into account numerical values for $d_{1}^{\left(1\right)}$ and $d_{2}^{\left(1\right)}$, we obtain the following values of the parameters $c_{jk}^{\left(1\right)}$:

(21) $$\begin{array}{c} c_{11}^{\left(1\right)}\approx 1.5013145387528147176730252470373223,\\ c_{21}^{\left(1\right)}\approx 0.50043817958427157255767508234577407,\\ c_{12}^{\left(1\right)}\approx 1.5000008642589575005473878767725752,\\ c_{22}^{\left(1\right)}\approx c_{21}^{\left(1\right)}\cdot 0.99912498383253616899527502360939620. \end{array}$$

This large number of digits, which is much higher than that needed for the single-precision computations, will be useful later in the case of higher precision.

Thus, finally, we obtained a new algorithm *InvSqrt1* that has the same structure as *InvSqrt*, but with different values of numerical coefficients (see [32]). In the case of two iterations, the code InvSqrt1 has more algebraic operations (one additional multiplication) in comparison to *InvSqrt*.

### 2.2. InvSqrt2 Algorithm

Assuming $a_{1}=a_{2}=1$ and $b_{1}=b_{2}=0$, we transform (12) into

(22) $$\begin{array}{c} {\tilde{y}}_{1}^{\left(2\right)}=\frac{1}{2}{\tilde{y}}_{0}^{\left(2\right)}\left(3-{\left({\tilde{y}}_{0}^{\left(2\right)}\right)}^{2}\;\tilde{x}\right)+\frac{1}{2}d_{1}{\tilde{y}}_{0}^{\left(2\right)},\\ {\tilde{y}}_{2}^{\left(2\right)}=\frac{1}{2}{\tilde{y}}_{1}^{\left(2\right)}\left(3-{\left({\tilde{y}}_{1}^{\left(2\right)}\right)}^{2}\;\tilde{x}\right)+\frac{1}{2}d_{2}{\tilde{y}}_{1}^{\left(2\right)}, \end{array}$$

where ${\tilde{y}}_{1}^{\left(2\right)}$ and ${\tilde{y}}_{2}^{\left(2\right)}$ depend on $\tilde{x}$, *t*, $d_{1}$, and $d_{2}$.

Parameters $t=t_{1}^{\left(2\right)}$ and $d_{1}=d_{1}^{\left(2\right)}$ are determined by the minimization of $\left|\right|\Delta_{1}^{\left(2\right)}\left(\tilde{x},t\right)\left|\right|$. Then, the parameter $d_{2}=d_{2}^{\left(2\right)}$ is determined by the minimization of $\left|\right|\Delta_{2}^{\left(2\right)}\left(\tilde{x},t_{1}^{\left(2\right)}\right)\left|\right|$ (see Appendix A.1 for details). As a result, we get:

(23) $$d_{1}^{\left(2\right)}=1.75791023259\cdot 10^{-3}\;,\;d_{2}^{\left(2\right)}≃1.159352515\cdot 10^{-6}\;,$$

and

(24) $$t_{1}^{\left(2\right)}\equiv t^{\left(2\right)}\approx 3.73157124016\;,\;R^{\left(2\right)}=1597466888=0x5F376908\;.$$

The theoretical relative errors are given by

(25) $$\begin{array}{c} \Delta_{1\max}^{\left(2\right)}\equiv \left|\right|\Delta_{1}^{\left(2\right)}\left(\tilde{x},t_{1}^{\left(2\right)}\right)\left|\right|\approx 0.87908\cdot 10^{-3},\\ \Delta_{2\max}^{\left(2\right)}\equiv \left|\right|\Delta_{2}^{\left(2\right)}\left(\tilde{x},t_{1}^{\left(2\right)}\right)\left|\right|\approx 5.80\cdot 10^{-7}. \end{array}$$

The coefficients in (13) are given by

(26) $$c_{1k}^{\left(2\right)}=\frac{3+d_{k}^{\left(2\right)}}{2}\;,\;c_{2k}^{\left(2\right)}=\frac{1}{2}\;.$$

Taking into account the numerical values for $d_{1}^{\left(2\right)}$ and $d_{2}^{\left(2\right)}$ (see (23)), we obtain the following values of the parameters $c_{jk}^{\left(2\right)}$:

(27) $$\begin{array}{c} c_{11}^{\left(2\right)}\approx 1.5008789551163345746409291568502392,\\ c_{12}^{\left(2\right)}\approx 1.5000005796762576644996810350809289,\\ c_{21}^{\left(2\right)}=c_{22}^{\left(2\right)}=0.5, \end{array}$$

where the large number of digits will be useful later in the case of higher precision. Thus, we completed the derivation of the code InvSqrt2:

*1.* **float***InvSqrt2*(**float** x){

*2.*   **float** halfx = 0.5f*x;

*3.*   **int** i = *(int*) &x;

*4.*   i = 0x5F376908 - (i>>1);

*5.*   **float** y = *(float*) &i;

*6.*   y* = 1.50087896f - halfx*y*y;

*7.*   y* = 1.50000057f - halfx*y*y;

*8.*   **return** y;

*9.* }

The code *InvSqrt2* contains a new magic constant ($R^{\left(2\right)}$) and has two lines (6 and 7) that were modified in comparison with the code InvSqrt. We point out that InvSqrt2 has the same number of algebraic operations as InvSqrt.

### 2.3. InvSqrt3 Algorithm

Now, we consider the algorithm (13) in its most general form:

(28) $$\begin{array}{c} {\tilde{y}}_{1}^{\left(3\right)}=k_{1}{\tilde{y}}_{0}^{\left(3\right)}\left(k_{2}-\tilde{x}{\left({\tilde{y}}_{0}^{\left(3\right)}\right)}^{2}\right),\\ {\tilde{y}}_{2}^{\left(3\right)}=k_{3}{\tilde{y}}_{1}^{\left(3\right)}\left(k_{4}-\tilde{x}{\left({\tilde{y}}_{1}^{\left(3\right)}\right)}^{2}\right), \end{array}$$

where $k_{j}$, $k_{2}$, $k_{3}$, and $k_{4}$ are constant. In Appendix A.2, we determine parameters $t=t_{1}^{\left(3\right)}$, $k_{1}$, and $k_{2}$ by minimization of $\left|\right|\Delta_{1}^{\left(3\right)}\left(\tilde{x},t\right)\left|\right|$. Then, the parameters $k_{3}$ and $k_{4}$ are determined by minimization of $\left|\right|\Delta_{2}^{\left(3\right)}\left(\tilde{x},t_{1}^{\left(3\right)}\right)\left|\right|$. As a result, we get:

(29) $$\begin{array}{c} k_{1}\approx 0.70395201\;,\;k_{2}\approx 2.3892451\;,\\ k_{3}\approx 0.50000005\;,\;k_{4}\approx 3.0000004\;, \end{array}$$

and

(30) $$t_{1}^{\left(3\right)}\equiv t^{\left(3\right)}=3,\;\;R^{\left(3\right)}=1595932672=0x5F200000\;.$$

The theoretical relative errors are given by

(31) $$\begin{array}{c} \Delta_{1\max}^{\left(3\right)}\equiv \left|\right|\Delta_{1}^{\left(3\right)}\left(\tilde{x},t_{1}^{\left(3\right)}\right)\left|\right|\approx 0.65007\cdot 10^{-3},\\ \Delta_{2\max}^{\left(3\right)}\equiv \left|\right|\Delta_{2}^{\left(3\right)}\left(\tilde{x},t_{1}^{\left(3\right)}\right)\left|\right|\approx 3.17\cdot 10^{-7}. \end{array}$$

**Figure 1:**
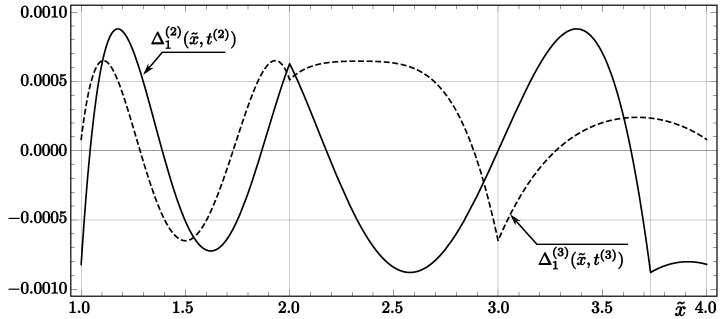
Theoretical relative errors of the first correction for the codes *InvSqrt2* and *InvSqrt3*. The solid line represents the function $\Delta_{1}^{\left(2\right)}\left(\tilde{x},t^{\left(2\right)}\right)$, while the dashed line represents $\Delta_{1}^{\left(3\right)}\left(\tilde{x},t^{\left(3\right)}\right)$.

They are significantly smaller (by 26% and 45%, respectively) than the analogous errors for *InvSqrt1* and *InvSqrt2* (see (19) and (25)). The comparison of error functions for *InvSqrt2* and *InvSqrt3* (in the case of one correction) is presented in Figure 1.

The numerical values of coefficients $c_{ij}^{\left(3\right)}$ (compare with (13)) are given by:

(32) $$\begin{array}{c} c_{11}^{\left(3\right)}=k_{1}k_{2}\approx 1.68191390868723079,\\ c_{21}^{\left(3\right)}=k_{1}\approx 0.703952009104829370,\\ c_{12}^{\left(3\right)}=k_{3}k_{4}\approx 1.50000036976749938,\\ c_{22}^{\left(3\right)}=k_{3}\approx 0.500000052823927419. \end{array}$$

Thus, we obtained the following code, called *InvSqrt3*: 

*1.* **float***InvSqrt3*(**float** x){

*2.*   **int** i = *(int*) &x;

*3.*   i = 0x5F200000 - (i>>1);

*4.*   **float** y = *(float*) &i;

*5.*   y* = 1.68191391f - 0.703952009f*x*y*y;

*6.*   y* = 1.50000036f - 0.500000053f*x*y*y;

*7.*   **return** y;

*8.* }

The code *InvSqrt3* has the same number of multiplications as *InvSqrt1*, which means that it is slightly more expensive than *InvSqrt* and *InvSqrt2*.

## 3. Generalizations

The codes presented in Section 2 can only be applied to normal numbers (1) of the type **float**. In this section, we show how to extend these results to subnormal numbers and to higher-precision formats.

### 3.1. Subnormal Numbers

Subnormal numbers are smaller than any normal number of the form of (1). In the single-precision case, positive subnormals can be represented as $m_{x}\cdot 2^{-126}$, where $m_{x}\in \left(0,1\right)$. They can also be characterized by nine first bits equal to zero (which also includes the case where x=0). In order to identify subnormals, we will make a bitwise conjunction (AND) of a given number with the integer 0x7f800000, which has all eight exponent bits equal to 1 and all 23 mantissa bits equal to 0. This bitwise conjunction is zero if and only if the given number is subnormal (including 0).

In the case of the single precision, the multiplication by $2^{24}$ transforms any subnormal number into a normal number. Therefore, we make this transformation; then, we apply one of our algorithms and, finally, make the inverse transformation (i.e., multiplying the result by $2^{-12}$). Thus, we get an approximated value of the inverse square root of the subnormal number. Note that $2^{24}$ is the smallest power of 2 with an even exponent that transforms all subnormals into normal numbers.

In the case of *InvSqrt3*, the procedure described above can be written in the form of the following code.

*1.* **float***InvSqrt3*s(**float** x){

*2.*   **int** i = *(int*) &x;

*3.*   **int** k = i & 0x7f800000;

*4.*   if (k==0) {

*5.*    x = 16777216.f*x; //16777216.f=pow(2.0f, 24)

*6.*    i = *(int*) &x;

*7.*   }

*8.*   i = 0x5F200000 - (i>>1);

*9.*   **float** y = *(float*) &i;

*10.*   y* = 1.68191391f - 0.703952009f*x*y*y;

*11.*   y* = 1.50000036f - 0.500000053f*x*y*y;

*12.*   if (k==0) **return** 4096.f*y; //4096.f=pow(2.0f, 12)

*13.*   **return** y;

*14.* }

The maximum relative errors for this code are presented in Section 4 (see Table 1).

### 3.2. Higher Precision

The above analysis was confined to the single-precision floating-point format. This is sufficient for many applications (especially microcontrollers), although the double-precision format is more popular. A trade-off between accuracy, computational cost, and memory usage is welcome [33]. In this subsection, we extend our analysis to double- and higher-precision formats. The calculations are almost the same. We just have to compute all involved constants with an appropriate accuracy. Low-bit arithmetic cases could be treated in exactly the same way. In this paper, however, we are focused on increasing the accuracy and on possible applications in distributed systems, so only the cases of higher precision are explicitly presented.

We present detailed results for double precision and some results (magic constants) for quadruple precision. Performing computations in **C**, we use the GCC Quad-Precision Math Library (working with numbers of type **_float128**). The crucial point is to express the magic constant *R* through the corresponding parameter *t*, which can be done with the formula:

(33) $$R=N_{m}\;\left(3B-1\right)/2+\left\lfloor N_{m}\left(t-2\right)/4-\mu_{\tilde{x}}/2\right\rceil$$

where $\mu_{\tilde{x}}\in \left\{0,1\right\}$, *t* depends on the considered algorithm and $N_{m}$ and *B* depend on the precision format used. Namely,

(34) $$\begin{array}{ccc} \mathrm{Sin}\mathrm{gle}\;\mathrm{precision}\;(32\text{-bit}): & \;N_{m}=2^{23}, & \;B=2^{7}-1\;,\\ \mathrm{Double}\;\mathrm{precision}\;(64\text{-bit}): & \;N_{m}=2^{52}, & \;B=2^{10}-1\;,\\ \mathrm{Quadruple}\;\mathrm{precision}\;(128\text{-bit}): & \;N_{m}=2^{112}, & \;B=2^{14}-1\;. \end{array}$$

In the case of the zeroth approximation (without Newton–Raphson corrections), the parameter *t* is given by:

(35) $$t_{0}=3.7309795598377727818740863479840422,$$

which can be compared with [31]. The corresponding magic constants computed from the formula (33) read:

(36) $$\begin{array}{c} \;32\mathrm{-bit}:\;R^{\left(0\right)}=0x5F37642F\\ \;64\mathrm{-bit}:\;R^{\left(0D\right)}=0x5FE6EC85E7DE30DA\\ 128\mathrm{-bit}:\;R^{\left(0Q\right)}=0x5FFE6EC85E7DE30DAABC602711840B0F. \end{array}$$

In this paper, we focus on the case of Newton–Raphson corrections, where the value of the parameter *t* may depend on the algorithm. For *InvSqrt* and *InvSqrt1*, we have:

(37) $$t_{1}^{\left(0\right)}=t_{1}^{\left(1\right)}=3.7298003391605705687151317499871860,$$

(see (Section 2.1); compare with [31,32]). Then, (33) yields the following magic constants:

(38) $$\begin{array}{c} \;32\mathrm{-bit}:\;R^{\left(1\right)}=0x5F375A86\\ \;64\mathrm{-bit}:\;R^{\left(1D\right)}=0x5FE6EB50C7B537A9\\ 128\mathrm{-bit}:\;R^{\left(1Q\right)}=0x5FFE6EB50C7B537A9CD9F02E504FCFC0. \end{array}$$

Actually, the above value of *R* in the 64-bit case (i.e., $R^{\left(1D\right)}$) corresponds to $\mu_{\tilde{x}}=1$ (the same value of *R* was obtained by Robertson for *InvSqrt*[24] with a different method). For $\mu_{\tilde{x}}=0$, we got an *R* greater by 1 (other results reported in this section do not depend on $\mu_{\tilde{x}}$). In the 128-bit case, Robertson obtained an *R* that was 1 less than our value (i.e., $R^{\left(1Q\right)}$).

In the case of *InvSqrt2*, we have

(39) $$t^{\left(2\right)}=3.7315712401613957182292407381942955$$

(compare with (A11)), which yields:

(40) $$\begin{array}{c} \;32\mathrm{-bit}:\;R^{\left(2\right)}=0x5F376908\\ \;64\mathrm{-bit}:\;R^{\left(2D\right)}=0x5FE6ED2102DCBFDA\\ 128\mathrm{-bit}:\;R^{\left(2Q\right)}=0x5FFE6ED2102DCBFDA59415059AC483B5. \end{array}$$

Finally, for *InvSqrt3*, we obtained:

(41) $$t^{\left(3\right)}=3\;$$

(see (A29)). The corresponding magic constants are given by:

(42) $$\begin{array}{c} \;32\mathrm{-bit}:\;R^{\left(3\right)}=0x5F200000\\ \;64\mathrm{-bit}:\;R^{\left(3D\right)}=0x5FE400000000000C\\ 128\mathrm{-bit}:\;R^{\left(3Q\right)}=0x5FFE4000000000000000000000000000. \end{array}$$

The parameters of the modified Newton–Raphson corrections for the higher-precision codes can be computed from the theoretical formulas used in the single-precision cases, taking into account an appropriate number of significant digits. In numerical experiments, we tested the algorithms *InvSqrt1D*, *InvSqrt2D*, and *InvSqrt3D* with the magic constants $R^{\left(1D\right)}$, $R^{\left(2D\right)}$, and $R^{\left(3D\right)}$, respectively, and the following coefficients in the modified Newton–Raphson iterations (compare with (21), (27), and (32), respectively):

(43) $$\begin{array}{c} c_{11}^{\left(1D\right)}=1.50131453875281472,\\ c_{21}^{\left(1D\right)}=0.500438179584271573,\\ c_{12}^{\left(1D\right)}=1.50000086425895750,\\ c_{22}^{\left(1D\right)}=c_{21}^{\left(1D\right)}\cdot 0.999124983832536169. \end{array}$$

(44) $$\begin{array}{c} c_{11}^{\left(2D\right)}=1.50087895511633457,\\ c_{12}^{\left(2D\right)}=1.50000057967625766,\\ c_{21}^{\left(2D\right)}=c_{22}^{\left(2D\right)}=0.5, \end{array}$$

(45) $$\begin{array}{c} c_{11}^{\left(3D\right)}=1.68191390868723079,\\ c_{21}^{\left(3D\right)}=0.703952009104829370,\\ c_{12}^{\left(3D\right)}=1.50000036976749938,\\ c_{22}^{\left(3D\right)}=0.500000052823927419. \end{array}$$

The algorithm *InvSqrt* and its improved versions are usually implemented in the single-precision case with no more than two Newton–Raphson corrections. However, in the case of higher precision, higher accuracy of the result is welcome. Then, a higher number of modified Newton–Raphson iterations could be considered. As an example, we present the algorithm *InvSqrt2D* with four iterations:

*1.* **double***InvSqrt2D*(**double** x){

*2.*   **double** halfx=0.5*x;

*3.*   **long long** i=*(long long*) &x;

*4.*   i=0x5FE6ED2102DCBFDA - (i>>1);

*5.*   **double** y =*(double*) &i;

*6.*   y* = 1.50087895511633457 - halfx*y*y;

*7.*   y* = 1.50000057967625766 - halfx*y*y;

*8.*   y* = 1.5000000000002520 - halfx*y*y;

*9.*   y* = 1.5000000000000000 - halfx*y*y;

*10.*   **return** y;

*11.* }

By removing Line *9*, we obtain the code *InvSqrt2D* with three iterations, and by also removing Line *8*, we get the code defined by (44). The maximum relative errors for this code are presented in Section 4 (see (52)).

## 4. Numerical Experiments

The numerical tests for the codes derived and presented in this paper were performed on an Intel Core i5-3470 processor using the TDM-GCC 4.9.2 32-bit compiler (when repeating these tests on the Intel i7-5700 processor, we obtained the same results, and comparisons with some other processors and compilers are given in Appendix B). In this section, we discuss round-off errors for the algorithms *InvSqrt2* and *InvSqrt3* (the case of single precision and two Newton–Raphson iterations) and then present the final results of analogous analysis for other codes described in this paper.

**Figure 2:**
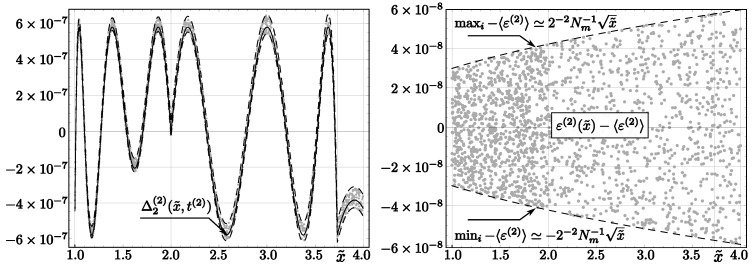
Theoretical and rounding errors of the code *InvSqrt2* (with two Newton–Raphson corrections). Left: The solid line represents $\Delta_{2}^{\left(2\right)}\left(\tilde{x},t^{\left(2\right)}\right)$, dashed lines correspond to $\Delta_{2}^{\left(2\right)}\left(\tilde{x},t^{\left(2\right)}\right)\pm 2^{-2}N_{m}^{-1}\sqrt{\tilde{x}}+\langle \varepsilon^{\left(2\right)}\rangle$, and dots represent errors for 4000 floating-point numbers *x* randomly chosen from the interval $\left(2^{-126},2^{128}\right)$. Right: relative error $\varepsilon^{\left(2\right)}$ (see (47)). Dashed lines correspond to the minimum and maximum values of these errors, and dots denote errors for 2000 values $\tilde{x}$ randomly chosen from the interval [1,4).

**Figure 3:**
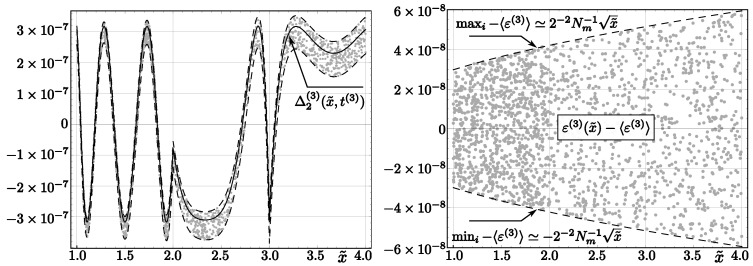
Theoretical and rounding errors of the code *InvSqrt3* (with two Newton–Raphson corrections). Left: The solid line represents $\Delta_{2}^{\left(3\right)}\left(\tilde{x},t^{\left(3\right)}\right)$, dashed lines correspond to $\Delta_{2}^{\left(3\right)}\left(\tilde{x},t^{\left(3\right)}\right)\pm 2^{-2}N_{m}^{-1}\sqrt{\tilde{x}}+\langle \varepsilon^{\left(3\right)}\rangle$, and dots represent errors for 4000 floating-point numbers *x* randomly chosen from the interval $\left(2^{-126},2^{128}\right)$. Right: relative error $\varepsilon^{\left(3\right)}$. Dashed lines correspond to minimum and maximum values of these errors, and dots denote errors for 2000 values $\tilde{x}$ randomly chosen from the interval [1,4).

Applying algorithms *InvSqrt2* and *InvSqrt3*, we obtain relative errors that differ slightly, due to round-off errors, from their analytical values (see Figure 2 and Figure 3; compare with [32] for an analogous discussion concerning *InvSqrt1*). Although we present only randomly chosen values in the figures, calculations were done for all **float** numbers *x* such that $e_{x}\in \left[-126,128\right)$.

The errors of numerical values returned by InvSqrt2

(46) $$\Delta_{2;N}^{\left(2\right)}\left(x\right)=\mathrm{sqrt}\left(x\right)*\mathrm{InvSqrt}2\left(x\right)-1\;$$

belong (for $e_{x}\neq -126$) to the interval $\left(-6.21\cdot 10^{-7},6.53\cdot 10^{-7}\right)$. For $e_{x}=-126$, we get a wider interval: $\left[-6.46\cdot 10^{-7},6.84\cdot 10^{-7}\right]$. These errors differ from the errors of ${\tilde{y}}_{2}^{\left(3\right)}\left(\tilde{x},t^{\left(2\right)}\right)$, which were determined analytically (compare (25)). We define:

(47) $$\varepsilon^{\left(2\right)}\left(\tilde{x}\right)=\frac{\mathrm{InvSqrt}2\left(x\right)-{\tilde{y}}_{2}^{\left(2\right)}\left(\tilde{x},t^{\left(2\right)}\right)}{{\tilde{y}}_{2}^{\left(2\right)}\left(\tilde{x},t^{\left(2\right)}\right)}.$$

This function, representing the observed blur of the **float** approximation of the *InvSqrt2* output, is symmetric with respect to its mean value

(48) $$\langle \varepsilon^{\left(2\right)}\rangle =2^{-1}N_{m}^{-1}\sum_{x\in [1,4)}\varepsilon^{\left(2\right)}\left(\tilde{x}\right)=1.636\cdot 10^{-8}$$

(see the right part of Figure 2), and covers the following range of values:

(49) $$\varepsilon^{\left(2\right)}\left(\tilde{x}\right)\in \left[-4.333\cdot 10^{-8},7.596\cdot 10^{-8}\right]\;.$$

Analogous results for the code *InvSqrt3* read:

(50) $$\langle \varepsilon^{\left(3\right)}\rangle =2^{-1}N_{m}^{-1}\sum_{x\in [1,4)}\varepsilon^{\left(3\right)}\left(\tilde{x}\right)=-1.890\cdot 10^{-8}$$

(51) $$\varepsilon^{\left(3\right)}\left(\tilde{x}\right)\in \left[-7.850\cdot 10^{-8},4.067\cdot 10^{-8}\right]\;.$$

The results produced by the same hardware with a 64-bit compiler have a greater amplitude of the error oscillations as compared with the 32-bit case (also compare Appendix B).

The maximum errors for the code *InvSqrt* and all codes presented in the previous sections are given in Table 2 (for codes with just one Newton–Raphson iteration) and Table 3 (the same codes but with two iterations).

Looking at the last column of Table 2 (this is the case of one iteration), we see that the code *InvSqrt1* is slightly more accurate than *InvSqrt2*, and both are roughly almost two times more accurate than *InvSqrt*. However, it is the code *InvSqrt3* that has the best accuracy. The computational costs of all these codes are practically the same (four multiplications in every case).

In the case of two iterations (Table 3), the code *InvSqrt3* is the most accurate as well. Compared with *InvSqrt*, its accuracy is 12 times higher for single precision and 14.5 times higher for double precision. However, the computational costs of *InvSqrt1* and *InvSqrt3* (eight multiplications) are higher than the cost of *InvSqrt* (seven multiplications). Therefore, the code *InvSqrt2* has some advantage, as it is less accurate than *InvSqrt3* but cheaper. In the single-precision case the code *InvSqrt2* is 6.8 times more accurate than *InvSqrt*.

We point out that the round-off errors in the single-precision case significantly decrease the gain of the accuracy of the new algorithms as compared with the theoretical values, especially in the case of two Newton–Raphson corrections (compare the third and the last column of Table 3).

The range of errors in the case of subnormal numbers (using the codes described in Section 3.1) is shown in Table 1. One can easily see that the relative errors are similar—in fact, even slightly lower—than in the case of normal numbers.

Although the original *InvSqrt* code used only one Newton–Raphson iteration, and in this paper, we focus mostly on two iterations, it is worthwhile to also briefly consider the case of more iterations. Then, the increased computational cost is accompanied by increased accuracy. We confine ourselves to the code *InvSqrt2* (see the end of Section 3.2), which is less expensive than *InvSqrt3* (and the advantage of *InvSqrt2* increases with the number of iterations). In the double-precision case, the maximum error for three Newton–Raphson corrections is much lower, and the fourth correction yields the best possible accuracy.

(52) $$\begin{array}{c} \Delta_{1D,N}^{\left(2\right)}=0.87908\times 10^{-3}\;,\\ \Delta_{2D,N}^{\left(2\right)}=0.57968\times 10^{-6}\;,\\ \Delta_{3D,N}^{\left(2\right)}=2.5213\times 10^{-13}\;,\\ \Delta_{4D,N}^{\left(2\right)}=1.1103\times 10^{-16}\;. \end{array}$$

In the case of single precision, we already get the best possible accuracy for the third correction, given by adding the line y* = 1.5f - halfx*y*y as Line *8* in the code *InvSqrt2* (see Section 2.2).

(53) $$\begin{array}{c} \Delta_{1,N}^{\left(2\right)}=0.87916\times 10^{-3}\;,\\ \Delta_{2,N}^{\left(2\right)}=0.68363\times 10^{-6}\;,\\ \Delta_{3,N}^{\left(2\right)}=0.89367\times 10^{-7}\; \end{array}$$

The derivation of all numerical codes presented in this paper did not take rounding errors into account. Therefore, the best floating-point parameters can be slightly different from the rounding of the best real parameters, all the more so since the distribution of the errors is still not exactly symmetric (compare fourth and fifth columns in Table 2 and Table 3). The full analysis of this problem is much more difficult than the analogous analysis for the original *InvSqrt* code because we now have several parameters to be optimized instead of a single magic constant. At the same time, the increase in accuracy is negligible. Actually, much greater differences in the accuracy appear in numerical experiments as a result of using different devices (see Appendix B).

As an example, we present the results of an experimental search in the case of the code *InvSqrt3* with one Newton–Raphson correction (three parameters to be optimized). The modified Newton–Raphson coefficients are found to be

(54) $$c_{11\;num}^{\left(3\right)}=1.681911588f\;,\;c_{21\;num}^{\left(3\right)}=k_{1}=0.7039490938f\;.$$

**Figure 4:**
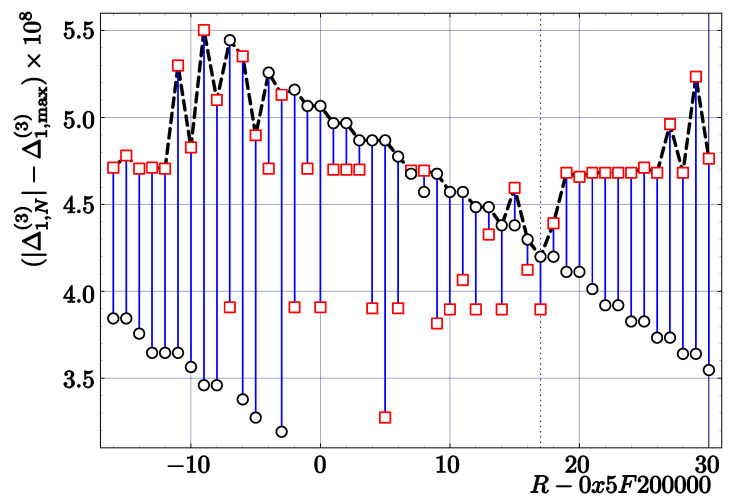
Maximum relative errors for the first Newton–Raphson correction in the code *InvSqrt3* as a function of *R* in the case of $k_{1}=0.7039490938f$ and $k_{1}k_{2}=1.681911588f$. Circles denote maximum errors ($\Delta_{1,N\max}^{\left(3\right)}$), while squares denote minimum errors ($|\Delta_{1,N\min}^{\left(3\right)}|$). The maximum error (shown by the dashed line) was determined by minimizing the maximum error for all floating-point numbers from [1,4).

Figure 4 summarizes the last step of this analysis. The dependence of maximum errors on *R* shows clearly that the optimum value for the magic constant is slightly shifted as compared to the theoretical (real) value:

(55) $$R_{num}^{\left(3\right)}=17+0x5f200000=0x5f200011\;.$$

The corresponding errors given by

(56) $$\Delta_{1,N\max}^{\left(3\right)}=6.50112284\cdot 10^{-4}\;,\;\Delta_{1,N\min}^{\left(3\right)}=-6.501092575\cdot 10^{-4}\;$$

are nearly symmetric. They are smaller than the maximum error $\Delta_{1,N}^{\left(3\right)}$ corresponding to our theoretical values, but only by about 0.001% (see Table 2).

## 5. Conclusions

We presented two new modifications (*InvSqrt2* and InvSqrt3) of the fast inverse square root code in single-, double-, and higher-precision versions. Each code has its own magic constant. All new algorithms are much more accurate than the original code *InvSqrt*. One of the new algorithms, *InvSqrt2*, has the same computational cost as *InvSqrt* in the case of any precision. Another code, *InvSqrt3*, has the best accuracy, but is more expensive if the number of Newton–Raphson corrections is greater than 1. However, its gain in accuracy is very high, even by more than 12 times for two iterations (see Table 3 in Section 4).

Our approach was to modify the Newton–Raphson method by introducing arbitrary parameters, which are then determined by minimizing the maximum relative error. It is expected that such modifications will provide a significant increase in accuracy, especially in the case of asymmetric error distribution for Newton–Raphson corrections (and this is the case with the inverse square root function when these corrections are non-positive). One has to remember that due to rounding errors, our theoretical results may differ from the best floating-point parameters, but the difference is negligible (see the end of Section 4). In fact, parameters (magic constants and modified Newton–Raphson coefficients) from a certain range near the values obtained in this article seem equally good for all practical purposes.

Concerning potential applications, we have to acknowledge that for general-purpose computing, the SSE and AVX reciprocal square root instructions are faster and more accurate. We hope, however, that the proposed algorithms can be applied in embedded systems and microcontrollers without a hardware floating-point divider, and potentially in FPGAs. Moreover, in contrast to the SSE and AVX instructions, our approach can be easily extended to computational platforms of high precision, like 256-bit or 512-bit platforms.

## Figures and Tables

**Table 1:** Relative numerical errors for the first and second corrections in the case of the type **float** (compiler 32-bit) for subnormal numbers.

Algorithm	$\Delta_{1,N\min}^{\left(i\right)}$	$\Delta_{1,N\max}^{\left(i\right)}$	$\Delta_{2,N\min}^{\left(i\right)}$	$\Delta_{2,N\max}^{\left(i\right)}$
*InvSqrt1*	$-0.87642\times 10^{-3}$	$0.87644\times 10^{-3}$	$-0.66221\times 10^{-6}$	$0.63442\times 10^{-6}$
*InvSqrt2*	$-0.87916\times 10^{-3}$	$0.87911\times 10^{-3}$	$-0.62060\times 10^{-6}$	$0.65285\times 10^{-6}$
*InvSqrt3*	$-0.65016\times 10^{-3}$	$0.65006\times 10^{-3}$	$-0.38701\times 10^{-6}$	$0.35196\times 10^{-6}$

**Table 2:** Relative numerical errors for the first correction in the case of type **float** (compiler 32-bit). In the case of type **double**, the errors are equal to theoretical errors $\pm \Delta_{1\max}^{\left(i\right)}$ up to the accuracy given in the table.

Algorithm	*i*	$\Delta_{1\max}^{\left(i\right)}$	$\Delta_{1,N\min}^{\left(i\right)}$	$\Delta_{1,N\max}^{\left(i\right)}$	$\Delta_{1,N}^{\left(i\right)}$
*InvSqrt*	0	$1.75118\times 10^{-3}$	$-1.75124\times 10^{-3}$	$0.00008\times 10^{-3}$	$1.75124\times 10^{-3}$
*InvSqrt1*	1	$0.87636\times 10^{-3}$	$-0.87642\times 10^{-3}$	$0.87645\times 10^{-3}$	$0.87645\times 10^{-3}$
*InvSqrt2*	2	$0.87908\times 10^{-3}$	$-0.87916\times 10^{-3}$	$0.87914\times 10^{-3}$	$0.87916\times 10^{-3}$
*InvSqrt3*	3	$0.65007\times 10^{-3}$	$-0.65017\times 10^{-3}$	$0.65006\cdot 10^{-3}$	$0.65017\times 10^{-3}$

**Table 3:** Relative numerical errors for the second correction in the case of type **float** (compiler 32-bit). In the case of type **double**, the errors are equal to theoretical errors $\pm \Delta_{2\max}^{\left(i\right)}$ up to the accuracy given in the table.

Algorithm	*i*	$\Delta_{2\max}^{\left(i\right)}$	$\Delta_{2,N\min}^{\left(i\right)}$	$\Delta_{2,N\max}^{\left(i\right)}$	$\Delta_{2,N}^{\left(i\right)}$
*InvSqrt*	0	$4.59728\times 10^{-6}$	$-4.65441\times 10^{-6}$	$0.08336\times 10^{-6}$	$4.65441\times 10^{-6}$
*InvSqrt1*	1	$0.57617\times 10^{-6}$	$-0.67207\times 10^{-6}$	$0.64871\times 10^{-6}$	$0.67207\times 10^{-6}$
*InvSqrt2*	2	$0.57968\times 10^{-6}$	$-0.64591\times 10^{-6}$	$0.68363\times 10^{-6}$	$0.68363\times 10^{-6}$
*InvSqrt3*	3	$0.31694\times 10^{-6}$	$-0.38701\times 10^{-6}$	$0.35198\times 10^{-6}$	$0.38701\times 10^{-6}$

## Data Availability

Data is contained within the article.

## Appendix A. Analytical Derivation of Modified Newton–Raphson Coefficients

### Appendix A.1. Algorithm InvSqrt2

We will determine the parameters $d_{1}$ and $d_{2}$ in formulas (22) that minimize the maximum error. Substituting (16) (with n=2) into (22), we get:

(A1) $$\Delta_{1}^{\left(2\right)}\left(\tilde{x},t,d_{1}\right)=\frac{1}{2}d_{1}\left(1+{\tilde{\delta }}_{0}\left(\tilde{x},t\right)\right)-\frac{1}{2}{\tilde{\delta }}_{0}^{2}\left(\tilde{x},t\right)\left(3+{\tilde{\delta }}_{0}\left(\tilde{x},t\right)\right)$$

(A2) $$\Delta_{2}^{\left(2\right)}\left(\tilde{x},t,d_{1},d_{2}\right)=\frac{1}{2}d_{2}\left(1+\Delta_{1}^{\left(2\right)}\right)-\frac{1}{2}{\left(\Delta_{1}^{\left(2\right)}\right)}^{2}\left(3+\Delta_{1}^{\left(2\right)}\right),$$

where $\Delta_{1}^{\left(2\right)}\equiv \Delta_{1}^{\left(2\right)}\left(\tilde{x},t,d_{1}\right)$ and ${\tilde{\delta }}_{0}\left(\tilde{x},t\right)$ is the relative error of the zeroth approximation (the function ${\tilde{\delta }}_{0}\left(\tilde{x},t\right)$ is presented and discussed in [31,32]).

First, we are going to determine the *t* and $d_{1}^{\left(2\right)}$ that minimize the maximum absolute value of the relative error of the first correction. We have to solve the following equation:

(A3) $$0=\frac{\partial \Delta_{1}^{\left(2\right)}\left(\tilde{x},t\right)}{\partial {\tilde{\delta }}_{0}\left(\tilde{x},t\right)}=\frac{1}{2}d_{1}^{\left(2\right)}-3{\tilde{\delta }}_{0}\left(\tilde{x},t\right)-\frac{3}{2}{\tilde{\delta }}_{0}^{2}\left(\tilde{x},t\right).$$

Its solution

(A4) $${\tilde{\delta }}^{+}=\sqrt{1+d_{1}^{\left(2\right)}/3}-1$$

corresponds to the value

(A5) $$\Delta_{1\max}^{\left(2\right)}=\frac{1}{2}d_{1}^{\left(2\right)}\left(1+{\tilde{\delta }}^{+}\right)-\frac{1}{2}{\tilde{\delta }}^{+2}\left(3+{\tilde{\delta }}^{+}\right),$$

which is a maximum of $\Delta_{1}^{\left(2\right)}\left(\tilde{x},t\right)$ because its second derivative with respect to $\tilde{x}$, i.e.,

(A6) $$\partial_{\tilde{x}}^{2}\Delta_{1}^{\left(2\right)}\left(\tilde{x},t\right)=\partial_{\tilde{x}}^{2}{\tilde{\delta }}_{0}\left(\tilde{x},t\right)\partial_{{\tilde{\delta }}_{0}}\Delta_{1}^{\left(2\right)}\left(\tilde{x},t\right)-3\left(1+{\tilde{\delta }}_{0}\left(\tilde{x},t\right)\right){\left(\partial_{x}{\tilde{\delta }}_{0}\left(\tilde{x},t\right)\right)}^{2},$$

is negative. In order to determine the dependence of $d_{1}^{\left(2\right)}$ on the parameter *t*, we solve the equation

(A7) $$-\Delta_{1}^{\left(2\right)}\left(t,t\right)=\Delta_{1\max}^{\left(2\right)},$$

which (for some $t=t_{1}^{\left(2\right)}$) equates the maximum value of error with the modulus of the minimum value of error. Thus, we obtain the following relations:

(A8) $$\begin{array}{cc} \delta^{+} & =-1-\frac{1}{4}\sqrt{t}+\frac{1}{8}\frac{t}{f(t)}+\frac{1}{2}f\left(t\right), \end{array}$$

(A9) $$\begin{array}{cc} d_{1}^{\left(2\right)} & =-3+\frac{9}{16}t+\frac{3}{64}t^{2}f^{-2}\left(t\right)-\frac{3}{16}t^{3/2}f^{-1}\left(t\right)-\frac{3}{4}\sqrt{t}f\left(t\right)+\frac{3}{4}f^{2}\left(t\right), \end{array}$$

where

$$f\left(t\right)={\left[8+t^{3/2}/8+4\sqrt{4+t^{3/2}/8}\right]}^{1/3}.$$

The last step consists in equating the minimum boundary value of the error of analyzed correction with its smallest local minimum:

(A10) $$\Delta_{1}^{\left(2\right)}\left(t,t\right)=\frac{1}{2}d_{1}^{\left(2\right)}\left(1+{\tilde{\delta }}_{0}\left({\tilde{x}}_{0}^{II},t\right)\right)-\frac{1}{2}{\tilde{\delta }}_{0}^{2}\left({\tilde{x}}_{0}^{II},t\right)\left(3+{\tilde{\delta }}_{0}\left({\tilde{x}}_{0}^{II},t\right)\right),$$

where $x_{0}^{II}=\left(4+t\right)/3$ (see [31]). Solving the Equation (A10) numerically, we obtain the following value of *t*:

(A11) $$t_{1}^{\left(2\right)}≃3.73157124016,$$

which corresponds to the following magic constant:

(A12) $$R^{\left(2\right)}=1597466888=0x5F376908\;.$$

Taking into account (A8), we compute

(A13) $$d_{1}^{\left(2\right)}=1.75791023259\cdot 10^{-3},$$

and, using (A4) and (A5), we get

(A14) $$\Delta_{1\max}^{\left(2\right)}≃8.7908386407\cdot 10^{-4}≃\frac{{\tilde{\delta }}_{1\max}}{1.99}.$$

In the case of the second correction, we keep the obtained value $t=t_{1}^{\left(2\right)}$ and determine the parameter $d_{2}^{\left(2\right)}$, which equates the maximum value of the error with the modulus of its global minimum. $\Delta_{2}^{\left(2\right)}\left(\tilde{x},t_{1}^{\left(2\right)}\right)$ is increasing (decreasing) with respect to negative (positive) $\Delta_{1}^{\left(2\right)}\left(\tilde{x},t_{1}^{\left(2\right)}\right)$ and has local minima that come only from positive maxima and negative minima. Therefore, the global minimum should correspond to the global minimum $-\Delta_{1\max}^{\left(2\right)}$ or to the global maximum $\Delta_{1\max}^{\left(2\right)}$. Substituting these values into Equation (A2) in the place of $\Delta_{1}^{\left(2\right)}\left(\tilde{x},t_{1}^{\left(2\right)}\right)$, we obtain that deeper minima of $\left(-\Delta_{2\max}^{\left(2\right)}\right)$ come from the global minimum of the first correction:

(A15) $$-\Delta_{2\max}^{\left(2\right)}=\frac{1}{2}d_{2}^{\left(2\right)}\left(1-\Delta_{1\max}^{\left(2\right)}\right)-\frac{1}{2}\Delta_{1\max}^{(2)2}\left(3-\Delta_{1\max}^{\left(2\right)}\right),$$

and the maximum, by analogy to the first correction, corresponds to the following value of $\Delta_{1}^{\left(2\right)}\left(\tilde{x},t_{1}^{\left(2\right)}\right)$:

(A16) $$\Delta^{+}=\sqrt{1+d_{2}^{\left(2\right)}/3}-1.$$

Solving the equation

(A17) $$\Delta_{2\max}^{\left(2\right)}=\frac{1}{2}d_{2}^{\left(2\right)}\left(1+\Delta^{+}\right)-\frac{1}{2}\Delta^{+2}\left(3+\Delta^{+}\right),$$

we get

(A18) $$d_{2}^{\left(2\right)}≃1.159352515\cdot 10^{-6}\;\mathrm{and}\;\Delta_{2\max}^{\left(2\right)}≃5.796763137\cdot 10^{-7}≃\frac{{\tilde{\delta }}_{2\max}}{7.93}.$$

### Appendix A.2. Algorithm InvSqrt3

Parameters $k_{1},k_{2},k_{3}$, and $k_{4}$ in the formula (28) will be determined by minimization of the maximum error. The relative error functions for (28) are given by:

(A19) $$\Delta_{j}^{\left(3\right)}=\sqrt{\tilde{x}}\;{\tilde{y}}_{j}^{\left(3\right)}-1,\;$$

where j=1,2. Substituting (A19) into (28), we obtain:

(A20) $$\begin{array}{c} \Delta_{1}^{\left(3\right)}\left(\tilde{x},t,k_{1},k_{2}\right)=k_{1}k_{2}\left({\tilde{\delta }}_{0}\left(\tilde{x},t\right)+1\right)-k_{1}{\left({\tilde{\delta }}_{0}\left(\tilde{x},t\right)+1\right)}^{3}-1\;,\\ \Delta_{2}^{\left(3\right)}\left(\tilde{x},t,k\right)=k_{3}k_{4}\left(\Delta_{1}^{\left(3\right)}\left(\tilde{x},t,k_{1},k_{2}\right)+1\right)-k_{3}{\left(\Delta_{1}^{\left(3\right)}\left(\tilde{x},t,k_{1},k_{2}\right)+1\right)}^{3}-1\;, \end{array}$$

where $k=(k_{1},k_{2},k_{3},k_{4})$ and ${\tilde{\delta }}_{0}\left(\tilde{x},t\right)$ is the relative error of the zeroth approximation (see [31,32]).

We are going to find parameters *t* and *k* such that the error functions take extreme values. We begin with $\Delta_{1}^{\left(3\right)}$.

(A21) $$\partial_{\tilde{x}}\Delta_{1}^{\left(3\right)}\left(\tilde{x},t,k_{1},k_{2}\right)=\left(k_{1}k_{2}-3k_{1}{\left({\tilde{\delta }}_{0}\left(\tilde{x},t\right)+1\right)}^{2}\right)\partial_{\tilde{x}}{\tilde{\delta }}_{0}\left(\tilde{x},t\right).$$

Therefore, the local extremes of $\Delta_{1}^{\left(3\right)}$ can be located either at the same points as the extremes of ${\tilde{\delta }}_{0}\left(\tilde{x},t\right)$ or at the $\tilde{x}$ satisfying the equation:

(A22) $${\tilde{\delta }}_{0}\left(\tilde{x},t\right)=\delta_{1}^{\left(e\right)}$$

where

(A23) $$\delta_{1}^{\left(e\right)}=\sqrt{k_{2}/3}-1\;.$$

The extremes corresponding to $\delta_{1}^{\left(e\right)}$ are global maxima equal to

(A24) $$\Delta_{1\max}^{\left(3\right)}=k_{1}k_{2}\left(\delta_{1}^{\left(e\right)}+1\right)-k_{1}{\left(\delta_{1}^{\left(e\right)}+1\right)}^{3}-1=2k_{1}{\left(\frac{k_{2}}{3}\right)}^{3/2}-1\;.$$

The two extremes of ${\tilde{\delta }}_{0}\left(\tilde{x},t\right)$, located at ${\tilde{x}}_{0}^{I}=\left(6+t\right)/6$ and ${\tilde{x}}_{0}^{II}=\left(4+t\right)/3$ (see [31]), can correspond either to minima or to maxima of $\Delta_{1}^{\left(3\right)}$ (depending on parameters *t*, $k_{1}$, and $k_{2}$). If ${\tilde{\delta }}_{0}\left({\tilde{x}}_{0}^{I/II},t\right)<\delta_{1}^{\left(e\right)}$, we have a maximum. The case ${\tilde{\delta }}_{0}\left({\tilde{x}}_{0}^{I/II},t\right)>\delta_{1}^{\left(e\right)}$ corresponds to a minimum.

Global extremes of $\Delta_{1}^{\left(3\right)}$ can be either local extremes or boundary values (which, in turn, correspond to global extremes of ${\tilde{\delta }}_{0}\left(\tilde{x},t\right)$). We recall that the global minimum of ${\tilde{\delta }}_{0}\left(\tilde{x},t\right)$ is at $\tilde{x}=t$ and the global maximum is at ${\tilde{x}}_{0}^{I}$ or ${\tilde{x}}_{0}^{II}$ [31]. Therefore, in order to find the parameters $k_{1}$ and $k_{2}$ that minimize the maximal value of $|\Delta_{1}^{\left(3\right)}|$, we have to solve two equations:

(A25) $$\Delta_{1\max}^{\left(3\right)}+\Delta_{1}^{\left(3\right)}\left(t,t,k_{1},k_{2}\right)=0,$$

(A26) $$\Delta_{1}^{\left(3\right)}\left({\tilde{x}}_{0}^{N},t,k_{1},k_{2}\right)=\Delta_{1}^{\left(3\right)}\left(t,t,k_{1},k_{2}\right),\left[2ex\right]$$

where N=I for $t\in (2,2^{5/3}+2^{4/3}-2)$ and N=II for $t\in (2^{5/3}+2^{4/3}-2,4)$. Note that $\tilde{\delta }\left({\tilde{x}}_{0}^{N},t\right)=\max\left\{\tilde{\delta }\left({\tilde{x}}_{0}^{I},t\right),\tilde{\delta }\left({\tilde{x}}_{0}^{II},t\right)\right\}$ and $\Delta_{1}^{\left(3\right)}\left({\tilde{x}}_{0}^{N},t,k_{1},k_{2}\right)$ is a minimum of $\Delta_{1}^{\left(3\right)}$.

The solution of the system (A25) and (A26) corresponds to the case N=I and is given by:

(A27) $$\left\{\begin{array}{c} k_{1}=2{\left(2\cdot 3^{-3/2}k_{2}^{3/2}+t^{1/2}k_{2}/2-t^{3/2}/8\right)}^{-1}\\ k_{2}=\left[{\left(1+t/6\right)}^{3}+\sqrt{t}{\left(1+t/6\right)}^{3/2}+t\right]/4\;. \end{array}\right.$$

Thus, $\Delta_{1\max}^{\left(3\right)}$, given by (A24), is a function of one variable *t*, and we can easily find its minimum value. It is enough to compute

(A28) $$\frac{d\;\Delta_{1\max}^{\left(3\right)}}{dt}\equiv \sqrt{\frac{k_{2}}{3}}\left(\frac{2}{3}k_{2}\frac{d\;k_{1}}{dt}+k_{1}\frac{d\;k_{2}}{dt}\right)=0\;.$$

We obtain $t=t_{1}^{\left(3\right)}$, where

(A29) $$t_{1}^{\left(3\right)}=3,\;\;R^{\left(3\right)}=1595932672=0x5F200000\;,$$

and, inserting $t=t_{1}^{\left(3\right)}$ into (A24) and (A27):

(A30) $$\Delta_{1\max}^{\left(3\right)}=\frac{-54\sqrt{3}-36\sqrt{6}+\sqrt{2}{\left(17+6\sqrt{2}\right)}^{3/2}}{54\sqrt{3}+36\sqrt{6}+\sqrt{2}{\left(17+6\sqrt{2}\right)}^{3/2}}≃6.5007\cdot 10^{-4},$$

(A31) $$\begin{array}{c} k_{1}=\frac{256}{54\sqrt{3}+36\sqrt{6}+12\sqrt{17+6\sqrt{2}}+17\sqrt{34+12\sqrt{2}}}\approx 0.7039520,\\ k_{2}=\frac{51+18\sqrt{2}}{32}\approx 2.3892451. \end{array}$$

In order to find the parameters $k_{3}$ and $k_{4}$ that minimize the maximal relative error of the second correction, we fix $t=t_{1}^{\left(3\right)}$. Then, we have to solve the following Equations (obtained in the same way as Equations (A25) and (A26)):

(A32) $$\left\{\begin{array}{cc} \Delta_{2}^{\left(3\right)}\left(t,t,k_{1},k_{2},k_{3},k_{4}\right)= & k_{3}k_{4}\left(\Delta_{1\max}^{\left(3\right)}+1\right)-k_{3}{\left(\Delta_{1\max}^{\left(3\right)}+1\right)}^{3}-1\;,\\ \Delta_{2}^{\left(3\right)}\left(t,t,k_{1},k_{2},k_{3},k_{4}\right)= & -k_{3}k_{4}\left(\delta_{2}^{\left(e\right)}+1\right)+k_{3}{\left(\delta_{2}^{\left(e\right)}+1\right)}^{3}+1\;, \end{array}\right.$$

where

(A33) $$\delta_{2}^{\left(e\right)}=\sqrt{k_{4}/3}-1\;$$

corresponds to $\Delta_{1}^{\left(3\right)}\left(\tilde{x},t,k_{1},k_{2}\right)$ satisfying:

$$0=\partial_{\Delta_{1}^{\left(3\right)}\left(\tilde{x},t,k_{1},k_{2}\right)}\Delta_{2}^{\left(3\right)}\left(\tilde{x},t,k_{1},k_{2},k_{3},k_{4}\right)=k_{3}k_{4}-3k_{3}{\left(\Delta_{1}^{\left(3\right)}\left(\tilde{x},t,k_{1},k_{2}\right)+1\right)}^{2}.$$

Solving the system (A32), we get:

(A34) $$\begin{array}{c} k_{4}=3+{\left(\Delta_{1\max}^{\left(3\right)}\right)}^{2}\;,\\ k_{3}=2{\left(\left(2-\Delta_{1\max}^{\left(3\right)}+\delta_{2}^{\left(e\right)}\right)\left(2-\delta_{2}^{\left(e\right)}\left(1+\Delta_{1\max}^{\left(3\right)}+\delta_{2}^{\left(e\right)}\right)+\Delta_{1\max}^{\left(3\right)}\right)\right)}^{-1}\;,\\ \Delta_{2\max}^{\left(3\right)}=k_{3}k_{4}\left(\delta_{2}^{\left(e\right)}+1\right)-k_{3}{\left(\delta_{2}^{\left(e\right)}+1\right)}^{3}-1\;. \end{array}$$

Then, using (A30) and (A33), we get numerical values:

(A35) $$k_{3}\approx 0.50000005\;,\;k_{4}\approx 3.0000004\;,\;\Delta_{2\max}^{\left(3\right)}\approx 3.16943579\cdot 10^{-7}\;.$$

One can easily see that the obtained error $\Delta_{2\max}^{\left(3\right)}$ is only about half (55%) of the error $\Delta_{2\max}^{\left(2\right)}$.

## Appendix B. Numerical Experiments Using Different Processors and Compilers

The accuracy of our codes depends, to some extent, on the devices used for testing. In Section 4, we limited ourselves to the Intel Core i5 with the 32-bit compiler. In this Appendix, we present, for comparison, data from other devices (Table A1 and Table A2). All data are for the type **float** (single precision).

The first two columns with data correspond to the Intel Core i5 with the 32-bit compiler (described, in more detail, in Section 4), the next two columns correspond to the same processor, but with the 64-bit compiler. Then, we have results (the same) for three microcontrollers: STM32L432KC and TM4C123GH6PM (ARM Cortex-M4), as well as STM32F767ZIT6 (ARM Cortex-M7). The last two columns contain results for the ESP32-D0WDQ5 system with two Xtensa LX6 microprocessors.

**Table A1:** The range of numerical errors for the first correction depending on the CPU used.

Code	Intel Core i5 (32-bit)		Intel Core i5 (64-bit)		ARM Cortex M4-7		ESP32	
	$10^{3}\Delta_{\min}$	$10^{3}\Delta_{\max}$	$10^{3}\Delta_{\min}$	$10^{3}\Delta_{\max}$	$10^{3}\Delta_{\min}$	$10^{3}\Delta_{\max}$	$10^{3}\Delta_{\min}$	$10^{3}\Delta_{\max}$
*InvSqrt1*	−0.87642	0.87644	−0.87646	0.87654	−0.87649	0.87656	−0.87646	0.87652
*InvSqrt2*	−0.87916	0.87911	−0.87922	0.87924	−0.87923	0.87927	−0.87921	0.87922
*InvSqrt3*	−0.65017	0.65006	−0.65029	0.65017	−0.65028	0.65018	−0.65025	0.65014

**Table A2:** The range of numerical errors for the second correction depending on the processor used.

Code	Intel Core i5 (32-bit)		Intel Core i5 (64-bit)		ARM Cortex M4-7		ESP32	
	$10^{6}\Delta_{\min}$	$10^{6}\Delta_{\max}$	$10^{6}\Delta_{\min}$	$10^{6}\Delta_{\max}$	$10^{6}\Delta_{\min}$	$10^{6}\Delta_{\max}$	$10^{6}\Delta_{\min}$	$10^{6}\Delta_{\max}$
*InvSqrt1*	−0.66221	0.63504	−0.75813	0.78832	−0.80341	0.81933	−0.75548	0.77074
*InvSqrt2*	−0.62060	0.65287	−0.70266	0.77609	−0.74198	0.81605	−0.69991	0.76667
*InvSqrt3*	−0.38701	0.35198	−0.48605	0.45363	−0.51755	0.48057	−0.47314	0.44122
